# Molecular and Biochemical Basis of Minocycline-Induced Hyperpigmentation—The Study on Normal Human Melanocytes Exposed to UVA and UVB Radiation

**DOI:** 10.3390/ijms22073755

**Published:** 2021-04-04

**Authors:** Jakub Rok, Zuzanna Rzepka, Justyna Kowalska, Klaudia Banach, Artur Beberok, Dorota Wrześniok

**Affiliations:** Department of Pharmaceutical Chemistry, Faculty of Pharmaceutical Sciences in Sosnowiec, Medical University of Silesia, Jagiellońska 4, 41-200 Sosnowiec, Poland; zrzepka@sum.edu.pl (Z.R.); jkowalska@sum.edu.pl (J.K.); kbanach@sum.edu.pl (K.B.); abeberok@sum.edu.pl (A.B.); dwrzesniok@sum.edu.pl (D.W.)

**Keywords:** minocycline, melanocyte, melanin, tyrosinase, *MITF*, UV radiation

## Abstract

Minocycline is a drug which induces skin hyperpigmentation. Its frequency reaches up to 50% of treated patients. The adverse effect diminishes the great therapeutic potential of minocycline, including antibacterial, neuroprotective, anti-inflammatory and anti-cancer actions. It is supposed that an elevated melanin level and drug accumulation in melanin-containing cells are related to skin hyperpigmentation. This study aimed to evaluate molecular and biochemical mechanism of minocycline-induced hyperpigmentation in human normal melanocytes, as well as the contribution of UV radiation to this side effect. The experiments involved the evaluation of cyto- and phototoxic potential of the drug using cell imaging with light and confocal microscopes as well as biochemical and molecular analysis of melanogenesis. We showed that minocycline induced melanin synthesis in epidermal melanocytes. The action was intensified by UV irradiation, especially with the UVB spectrum. Minocycline stimulated the expression of microphthalmia-associated transcription factor (*MITF*) and tyrosinase (*TYR*) gene. Higher levels of melanin and increased activity of tyrosinase were also observed in treated cells. Moreover, minocycline triggered the supranuclear accumulation of tyrosinase, similar to UV radiation. The decreased level of premelanosome protein PMEL17 observed in all minocycline-treated cultures suggests disorder of the formation, maturation or distribution of melanosomes. The study revealed that minocycline itself was able to enhance melanin synthesis. The action was intensified by irradiation, especially with the UVB spectrum. Demonstrated results confirmed the potential role of melanin and UV radiation minocycline-induced skin hyperpigmentation.

## 1. Introduction

World Health Organization defined an adverse drug reaction (ADR) as “a response to a drug which is noxious and unintended and which occurs at doses normally used in man for the prophylaxis, diagnosis, or therapy of disease, or the modifications of physiological function”. The majority of ADRs (about 75–80%) is predictable, non-immunologic, usually dose-dependent and related to the drug pharmacology. The rest of ADRs are idiosyncratic, unpredictable and they may be immune-mediated [1,2]. It was estimated that ADRs are the reason for about 5–6% of hospital admissions and up to 25% of primary care attendances [2,3,4]. Cutaneous adverse drug reactions are thought to be one of the most frequent. Skin-related ADRs require additional treatment, medical management and generate significant costs for the payer as well as for the service provider [5]. In addition to the above, they also lead to a decrease in patient comfort and the limitations of drug usage. Drug-induced hyperpigmentation belongs to the most commonly occurred skin-related ADRs. The effect is usually observed in areas exposed to the sun. However, discolorations appear also in nails, the conjunctiva in the eye and mucous membranes [6]. It was estimated that drugs were the reason for 10 to 20% of all cases of acquired hyperpigmentation. Although the changes of skin pigmentation often disappear after discontinuation of a harmful therapy, in some cases, the pigmentation may be quite severe and last for a long time or become permanent. This is a serious esthetic, cosmetic and therapeutic problem, especially if the hyperpigmentation is present on the patient’s face [7,8].

Minocycline is one of the drugs inducing cutaneous ADRs. It belongs to semisynthetic second-generation tetracycline antibiotics [9]. The second dimethylamine substituent at position 7 distinguishes the structure of minocycline. This structural feature causes some beneficial pharmacokinetic properties. The compound is more lipophilic than other tetracyclines and it is almost completely absorbed after oral administration. It was stated that food did not have an influence on minocycline C_max_ and AUC, unlike in the case of other tetracycline antibiotics [10]. The drug is also well-distributed in the organism and easily penetrates the blood–brain barrier as well as to other tissues, including the skin [10,11].

Minocycline is a broad-spectrum antibiotic. The drug is used for the treatment of various infectious diseases, e.g., acne vulgaris, skin and soft tissue infections, Lyme disease, gastrointestinal infections, sexually transmitted diseases and zoonotic infections [12]. In addition to the antibiotic action, minocycline has many valuable non-antimicrobial properties. The pharmacological activity of minocycline includes neuroprotective, cardioprotective, antioxidant, anti-inflammatory and anti-cancer effects [13,14,15,16,17]. The beneficial and pleiotropic actions of minocycline create an opportunity to use the drug, among others, in the therapy of chronic pain, multiple sclerosis, stroke, sarcoidosis and hyperkeratosis, as well as in the field of psychiatry [18,19,20,21,22,23].

However, the great therapeutic potential of minocycline may be diminished by its adverse effects. Skin hyperpigmentation is one of the most characteristic minocycline-induced ADRs. Its frequency ranges from 3 to 15% of patients treated with minocycline [24]. However, it is suggested that the real percentage is higher and may reach up to 50% [25]. Currently, there are four types of minocycline-induced hyperpigmentation: (i) type 1 is blue–black discoloration, probably caused by iron chelates of minocycline; (ii) type 2 is blue-grey pigmentation caused by deposition of quinone metabolites of minocycline; (iii) type 3 is a kind of hyperpigmentation on sun-exposed areas which is related to an increase in the amount of melanin; (iv) type 4 is similar to type 3 and involves preexisting scars [26,27]. The exact mechanisms of minocycline-induced pigmentation remain unknown, but in most cases, the reason is related to long-term therapy and drug accumulation [28]. It is worth emphasizing that the chemical nature of minocycline allows to assume that the drug creates complexes with melanin polymers, similar to other tetracyclines [29,30]. Drug-melanin complexes may contribute to the drug deposition in pigmented tissues and to increase the risk of drug-induced toxic effects.

Above all, melanin polymers play numerous beneficial roles, including photoprotective and cytoprotective. Melanin pigments tend to accumulate within skin cells, i.e., keratinocytes and melanocytes, in the perinuclear area as supranuclear “caps”. This location allows melanin to shield DNA as a physical barrier that scatters UV radiation [31]. In addition, the pigments protect cells against the harmful effects of free radicals, toxins, chemicals and metals. Melanin acts as an antioxidant. It scavenges and quenches many reactive oxygen species and free radicals. It also presents charge-transfer redox activity. Moreover, melanin serves as a chelating agent for ions and binding buffer for a variety of biomolecules as well as xenobiotics [32]. Melanin is produced by highly specialized cells, i.e., melanocytes. The synthesis and storage of melanin take place in organelles called melanosomes [33]. Synthesis of melanin (the Raper–Mason pathway) is a multistep oxidation process. The initial step of melanogenesis is the conversion of tyrosine to DOPAquinone by the key enzyme, i.e., tyrosinase (EC 1.14.18.1). DOPAquinone can react with sulfhydryl compounds, like cysteine and successively form cysteinyl-DOPAs, cysteinyl-DOPAquinones and benzothiazine intermediates. Finally, the polymerization process leads to the production of yellow–red pheomelanin. In turn, during eumelanogenesis, DOPAquinone is spontaneously converted to DOPAchrome. In the next step, 5,6-dihydroxyindole and 5,6-dihydroxyindole-2-carboxylic acid are produced as the effect of spontaneous decarboxylation and enzymatic reaction catalyzed by DOPAchrome tautomerase, respectively. Both intermediates are oxidized by tyrosinase to quinone derivatives and next all four compounds polymerize to brown-black eumelanin [34,35]. Regulation of melanin synthesis is very complex. Melanogenesis may be stimulated by UV radiation, as well as by many endocrine and paracrine factors, such as melanocortins, adrenocorticotropic hormone, endothelins, β-endorphin, catecholamines, histamine, eicosanoids, mast cell growth factor, sex steroids and vitamin D. In turn, intrinsic inhibitors of melanogenesis include serotonin, dopamine, acetylcholine as well as various cytokines and growth factors, such as IL-1, IL-6, INF-α, INF-γ, TNF-α, TNF-β or TGF-β1. All these factors influence intracellular signal transduction pathways and may regulate the expression of genes related to melanin synthesis [36].

Considering the above problems, there raises the question of the influence of minocycline on the melanogenesis process in human normal melanocytes. In addition, the contribution of UV radiation to minocycline-induced hyperpigmentation has not been known so far. The study also aimed to assess the cyto- and phototoxicity of minocycline to the pigmented cells.

## 2. Results

### 2.1. Minocycline Inhibits the Proliferation of Human Melanocytes

**Figure 1:**
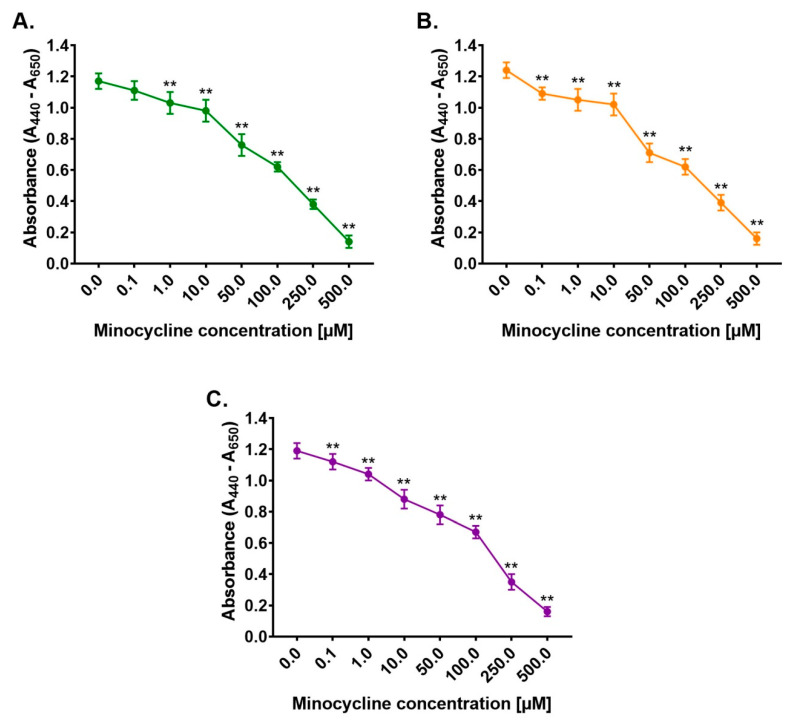
Minocycline inhibits proliferation of human normal melanocytes (**A**) non-irradiated (**B**) exposed to UVA (2 J/cm^2^) and (**C**) UVB (10 mJ/cm^2^) radiation. Cells were incubated with minocycline in concentrations ranging from 0.1 μM to 500 μM. Mean values ± SD from three independent experiments are presented. ** *p* < 0.005.

The general assessment of the cytotoxic and phototoxic action of minocycline was evaluated using Cell Proliferation Reagent WST-1. Human normal melanocytes were used in the study. The cells were treated with minocycline in concentrations ranging from 0.1 μM to 500 μM. Moreover, some samples were exposed to UVA and UVB radiation. The obtained results (Figure 1) indicated that the drug inhibited cell proliferation proportionally to a concentration. A statistically significant decrease was observed for concentrations from 1 µM to 500 µM. The highest concentration of minocycline decreased the absorbance by about 88% when compared to the control. Simultaneously, the applied dosages of UVA and UVB radiation did not influence cell proliferation significantly. Moreover, the results for irradiated cells pretreated with minocycline were similar to samples unexposed to the radiation. This indicated that minocycline had no phototoxic potential to melanocytes. The half-maximal effective concentrations for minocycline proved the conclusion. The EC_50_ values differed slightly and were calculated to be 98.7 µM, 94.4 µM and 103.9 µM for unirradiated, UVA-irradiated and UVB-irradiated cells, respectively. Based on the obtained results, the concentration of 100 μM was selected for the following experiments.

### 2.2. The Evaluation of Cell Number, Viability and Morphology of Human Melanocytes Treated with Minocycline and Exposed to UVA and UVB Radiation

**Figure 2:**
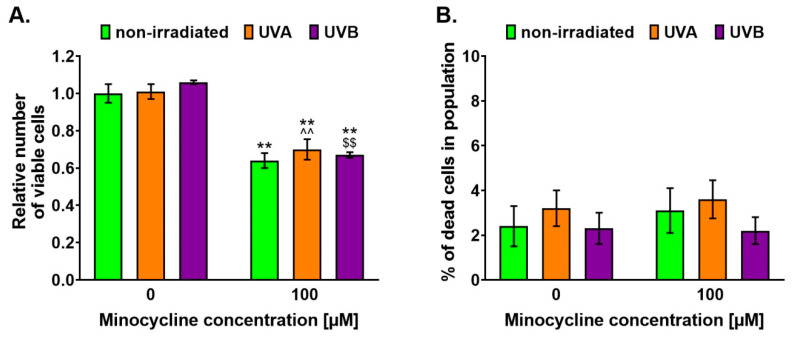
Minocycline inhibits the growth of human melanocytes but does not increase the percentage of dead cells. UVA and UVB radiation do not influence the effects. The results are presented as (**A**) the relative number of viable cells (**B**) and the percentage of dead cells in the tested population. ** *p* < 0.005 vs. non-irradiated cells; ^^ *p* < 0.005 vs. UVA-irradiated cells; $$ *p* < 0.005 vs. UVB-irradiated cells.

The estimation of melanocyte number and cell viability was conducted using fluorescence image cytometer NucleoCounter^®^ NC-3000™ (Figure 2). We showed that minocycline in a concentration of 100 μM decreased the cell number in treated cultures by about 36%. It was also noticed that the drug caused a decrease in irradiated cells by about 30% and 33% for UVA- and UVB-irradiated melanocytes, respectively. However, the applied dosages of UVA and UVB radiation did not influence the number of untreated cells. The analysis revealed that the viability of melanocytes was very similar in all tested probes and the number of dead cells was in the range of 2–4%, on average.

**Figure 3:**
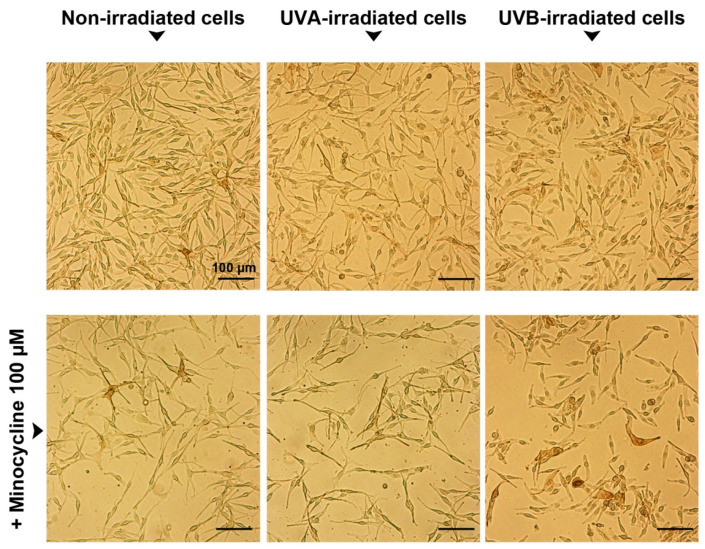
Microscopic evaluation of human normal melanocytes treated with 100 µM of minocycline and exposed to UVA and UVB radiation. Scale bar 100 μm.

The results obtained using the image cytometer were reflected in the microscopic analysis (Figure 3). The presented images indicated that the control culture as well as melanocytes exposed to UV radiation characterized high confluence of dendritic, attached and properly flattened cells. In turn, the treatment with minocycline caused a significant reduction of the cell number as well as induced changes in cell morphology. The biggest differences were found in a culture exposed to the drug and UVB radiation simultaneously. The melanocytes became thinner or shorter and some of them were spherical and swollen. Moreover, some highly pigmentated cells were observed in the culture.

### 2.3. Minocycline Stimulates the Expression of *MITF* and TYR Genes in Melanocytes Exposed to UVA and UVB Radiation

**Figure 4:**
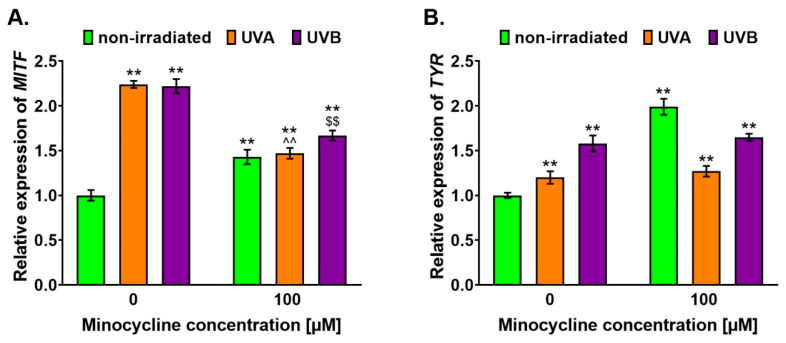
The influence of minocycline as well as UVA and UVB radiation on (**A**) *MITF* and (**B**) *TYR* expression in human normal melanocytes. The analysis was made using the RT-qPCR technique. *GAPDH* expression was used to normalize the obtained results. Transcriptional activity is presented as a relative expression level. ** *p* < 0.005 vs. non-irradiated cells; ^^ *p* < 0.005 vs. UVA-irradiated cells; $$ *p* < 0.005 vs. UVB-irradiated cells.

The gene expression of a transcription factor *MITF* and a key enzyme of melanogenesis, i.e., tyrosinase, were investigated. The analysis was performed in human melanocytes 6 h after the irradiation procedure. The results were expressed as a relative level of the control sample (Figure 4). It was found that minocycline as well as UVA and UVB radiation stimulated the expression of both tested genes. The highest level of *MITF* expression was observed in nontreated melanocytes exposed to UV radiation. The increase was similar for the UVA and UVB spectrum and was about 124% and 122%, respectively. The expression of *MITF* in all cell cultures treated with 100 µM of minocycline was elevated when compared to the control. However, the increase was significantly less than observed in irradiated melanocytes unexposed to the drug. The level of *MITF* after the treatment was found to be about 143, 147 and 167% for unirradiated melanocytes and irradiated with UVA and UVB, respectively.

In the case of *TYR* expression, the highest level (about 199%) was noticed for unirradiated melanocytes treated with the drug. However, the analysis indicated that minocycline had no significant effect on *TYR* expression in irradiated cells. The observed increase in *TYR* expression in untreated melanocytes exposed to UVA and UVB was about 20 and 60%, respectively.

### 2.4. Minocycline Increases Melanin Level in Human Melanocytes Irradiated with UVA and UVB

**Figure 5:**
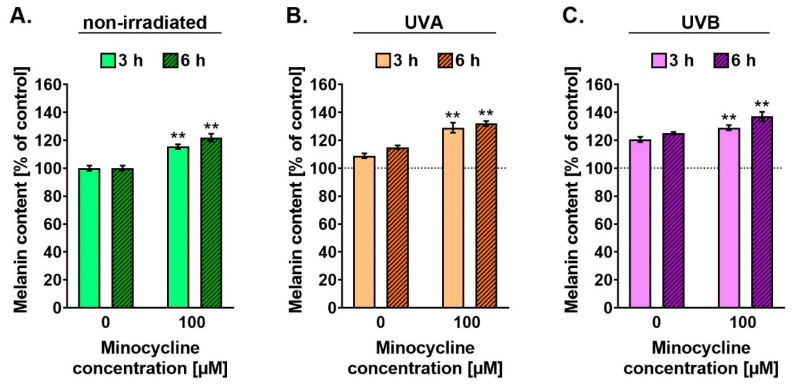
Minocycline causes an increase in melanin content in melanocytes (**A**) non-irradiated (**B**) exposed to UVA radiation (**C**) exposed to UVB radiation. Melanin content was normalized using total protein concentration. The results are presented as the percentage of control. ** *p* < 0.005.

The influence of minocycline and UV radiation on melanin content was investigated 3 h and 6 h after the irradiation. The obtained results (Figure 5) indicated that both tested factors, i.e., the drug and UV radiation, increased the melanin content proportionally to the time after the irradiation. It was found that minocycline and UVB radiation stimulated melanogenesis stronger than UVA radiation. Minocycline alone elevated melanin level by about 15 and 22% for 3 h and 6 h, respectively. In turn, the analogous results for untreated and UVB-irradiated cells were 21 and 26%. The melanin content in untreated and UVA-irradiated melanocytes was about 9 and 16% for 3 h and 6 h, respectively. The study revealed that none of the analyzed UV spectra intensified the minocycline effect significantly. The irradiation caused an increase in melanin content in melanocytes treated with minocycline in the range from 4 to 8% on average.

### 2.5. Minocycline Stimulates the Activity of Tyrosinase in Human Melanocytes Irradiated with UVA and UVB

**Figure 6:**
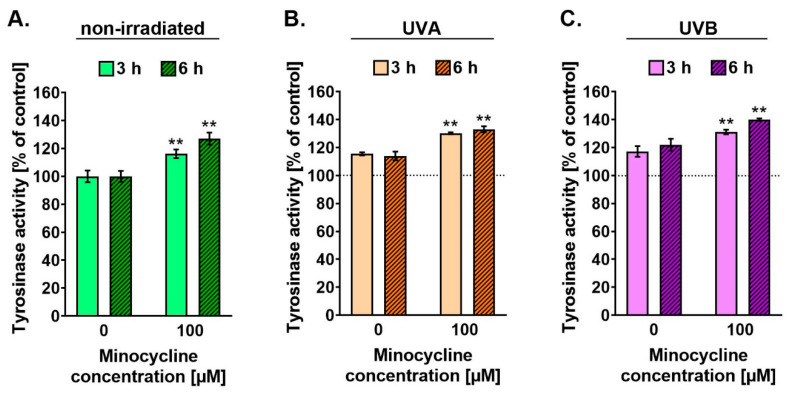
Minocycline causes an increase in the activity of tyrosinase in melanocytes (**A**) non-irradiated (**B**) exposed to UVA radiation (**C**) exposed to UVB radiation. The values for tyrosinase activity were normalized using total protein concentration. The results are presented as the percentage of control. ** *p* < 0.005.

The activity of tyrosinase was tested 3 h and 6 h after the cell irradiation. The profile of the obtained results (Figure 6) was similar to the results for melanin content. It was observed that minocycline as well as UVA and UVB radiation increased tyrosinase activity proportionally to the time after the irradiation. Minocycline and UVB radiation elevated the enzyme activity stronger than UVA radiation. The results after 6 h were about 127% and 123% for the drug and UVB spectrum, respectively. Moreover, it was found that pretreatment with minocycline increased the enzyme activity in melanocytes exposed to UVA and UVB radiation. The highest value (about 141%) was noticed for melanocytes treated with minocycline 6 h after UVB irradiation. Unlike the melanin level, the activity of tyrosinase was intensified by UVB radiation in minocycline-treated melanocytes. The difference between unexposed and UVB-irradiated cells treated with minocycline was about 20% after 6 h.

### 2.6. Minocycline Increases Level of *MITF* and Tyrosinase but Decreases Level of Pmel17 in Human Melanocytes Exposed to UVA and UVB Radiation

**Figure 7:**
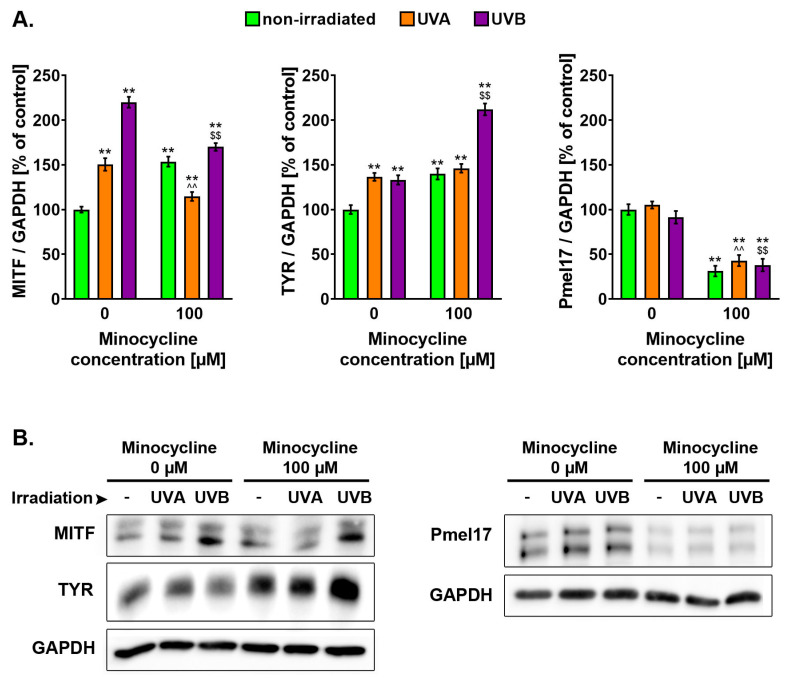
Minocycline as well as UVA and UVB radiation induce changes in the level of microphthalmia-associated transcription factor (*MITF*), tyrosinase (TYR) and premelanosome protein Pmel17 in normal melanocytes. The results of the Western blotting analysis are presented as (**A**) bar graph and (**B**) representative blot images. ** *p* < 0.005 vs. non-irradiated cells; ^^ *p* < 0.005 vs. UVA-irradiated cells; $$ *p* < 0.005 vs. UVB-irradiated cells.

The intracellular level of *MITF*, tyrosinase and Pmel17 protein was analyzed 6 h after the cell irradiation using Western blotting (Figure 7). It was found that minocycline and both UV spectra caused an increase in *MITF* and tyrosinase levels when compared to the control. The highest level of *MITF* (about 220%) was observed in untreated melanocytes exposed to UVB radiation. The results obtained for cells treated with minocycline and exposed to UVA radiation were about 150%. The level of *MITF* in irradiated cells pretreated with minocycline was significantly lower than in melanocytes unexposed to the drug. The decreases were about 36 and 50% for UVA and UVB-irradiated cells, respectively.

It was found that an increase in tyrosinase level was similar for cells treated with minocycline and exposed to UVA and UVB radiation. The results were about 137, 133 and 140%, respectively. The highest value (about 212%) was observed in samples exposed to the drug and UVB radiation. In turn, pretreatment with minocycline did not affect significantly the results for UVA-irradiated melanocytes.

The examination of Pmel17 indicated that the applied dosage of UVA and UVB radiation did not change the protein level in tested cells. The slight differences appeared to be statistically insignificant. However, a significant decrease in the protein content was observed in all probes treated with minocycline. The obtained results were about 31, 43 and 38% for unirradiated and exposed to UVA and UVB radiation, respectively.

### 2.7. Confocal Microscopy Analysis of Tyrosinase in Human Melanocytes Treated with Minocycline and Exposed to UVA and UVB Radiation

**Figure 8:**
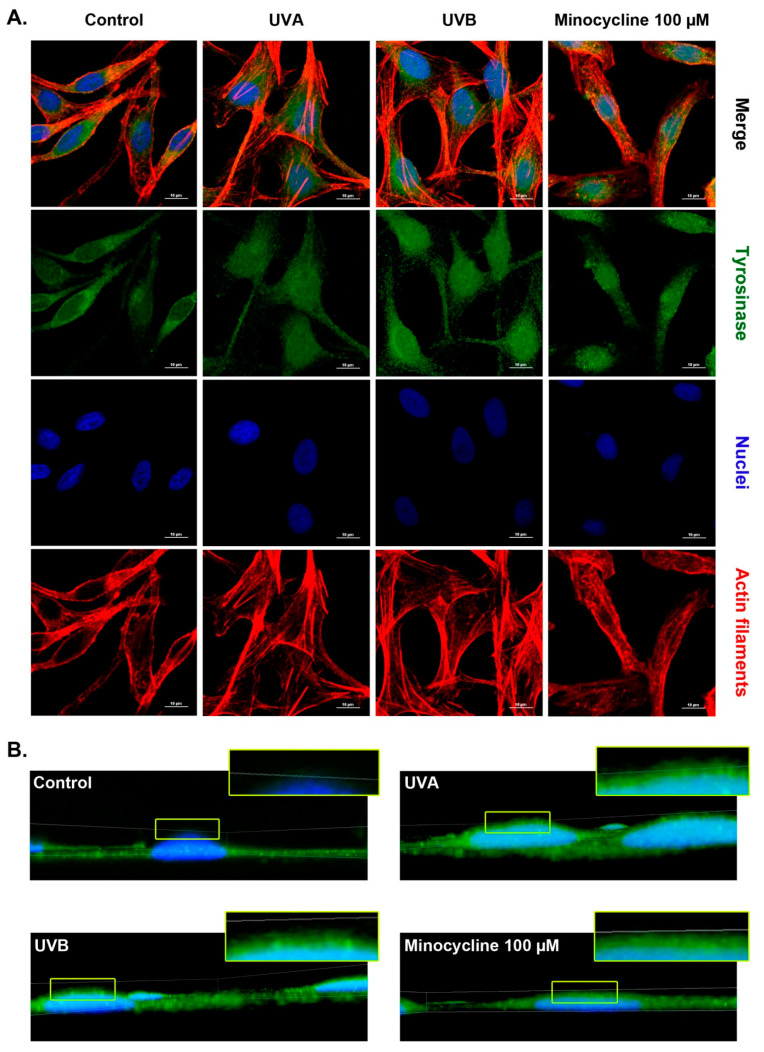
Confocal microscopy imaging of human normal melanocytes treated with minocycline and exposed to UVA and UVB radiation. (**A**) Photographs are presented as separate channels for tyrosinase, nuclei and actin filaments as well as merge images. Scale bar 100 μm. (**B**) 3D confocal imaging of normal melanocytes treated with minocycline and exposed to UVA or UVB radiation. The presented z-stack reconstructions show DAPI-stained nuclei (blue) and immunolabeled tyrosinase (green).

Confocal microscopy images (Figure 8A) presented darkly pigmented human melanocytes (i) non-irradiated and non-treated, (ii) exposed to UVA radiation, (iii) irradiated with UVB, (iv) treated with minocycline. Tested melanocytes were fixed 6 h after the cell irradiation. All cells were stained to visualize tyrosinase, nuclei and actin filaments. The picture analysis revealed changes in cell morphology. Most of the control cells were elongated and bipolar with two marked tapered extensions. Melanocytes exposed to UV radiation seemed to be more flattened, with a larger cell body when compared to the control. In turn, cells treated with minocycline remained their bipolar shape. However, their extensions were shorter and wider than observed in the control sample. The tested samples also differed in the intracellular distribution of tyrosinase. The analyzed protein tended to cumulate nearby nuclei of melanocytes in the control sample. In the case of cells treated with minocycline or exposed to UVB radiation, tyrosinase was situated mainly around the area of the nucleus, in the central part of melanocytes. On the other hand, the distribution of tyrosinase was more homogenous in all over the area of cells after UVA irradiation than in the rest of the tested samples.

Additionally, 3D confocal imaging (Figure 8B) showed the perinuclear localization of tyrosinase. It was found that the enzyme was generally localized in the basal plane of control melanocytes. In all other cases, tyrosinase was also observed over the nuclei. The finding indicated the formation of a kind of supranuclear caps.

## 3. Discussion

UV radiation is a well-known physical factor stimulating melanin synthesis. Melanogenesis is thought to be a protective mechanism against the harmful effects of the radiation. It has been found that about 50% of UVA (320–400 nm) and 9–15% of UVB (280-320 nm) radiation of sunlight reaches cells in the basal layer of the epidermis, including melanocytes [37]. The biological effects and functions of UV radiation are multiple. On the one hand, UV radiation takes part in body homeostasis. A physiological role of UV radiation involves its influence on chemical, hormonal and neural signalization as well as on vitamin D synthesis. It upregulates local neuroendocrine axes in the skin. The action depends on the release of cytokines, corticotropin-releasing hormone, proopiomelanocortin-peptides and enkephalins. Moreover, these factors may cause systemic effects after entering the circulation [38]. On the other hand, UV radiation may be responsible for skin pathology, including inflammation, photoaging, the formation of reactive oxygen species, cyclobutane pyrimidine dimers and pyrimidine (6–4) pyrimidone photoproducts, gene mutations, the arrest of cell cycle or cell death and activation of various adaptation mechanisms [39,40]. The exposure to UV is also responsible for the induction of drug-related phototoxic reactions.

Tetracyclines are one of the groups of drugs causing phototoxic reactions. The action is directly related to the antibiotic structure: the naphthacene nucleus and electron-dense regions along the lower peripheral region, which contribute to the absorption of UVA radiation and the generation of reactive oxygen species [41,42,43].

We showed that the applied dosages of UVA and UVB appeared to be non-phototoxic themselves. They did not influence cell proliferation and the percentage of dead cells in tested populations. In turn, minocycline significantly inhibited human darkly pigmented melanocyte proliferation in a concentration-dependent manner. However, the drug in a concentration of 100 µM (approximately EC_50_ value) did not affect the viability of cells. Moreover, the effect of minocycline was not changed by the irradiation. It is worth mentioning that lightly pigmented melanocytes (HEMn-LP) appeared to be more sensitive to minocycline. Recently published study indicated the EC_50_ value was two-times lower for lightly pigmented melanocytes then obtained for darkly pigmented cells [44]. The difference suggests that melanin may protect cells from toxic effects of minocycline. However, it is worth noting that 100 µM of minocycline did not influence the percentage of dead cells but significantly decreased cell number in tested populations of both types of melanocytes.

The previously conducted studies on human darkly pigmented melanocytes indicated that the cytotoxic effect of chlortetracycline, doxycycline, oxytetracycline and tetracycline was significantly enhanced by UVA radiation (1.3 J/cm^2^) [30,45,46,47]. In the case of chlortetracycline and oxytetracycline applied in the EC_50_ concentration, the irradiation increased the cytotoxic action by about 40%. A similar effect was not observed with minocycline, even at a higher dose of UVA radiation. The findings suggest that minocycline is much less phototoxic than other tetracyclines. The reason for this phenomenon may be related to the presence of 7-dimethylamine substituent, antioxidant properties and perhaps the ability to intensify melanogenesis. The studies cited above revealed that chlortetracycline, doxycycline, oxytetracycline and tetracycline did not augment melanin synthesis in non-irradiated melanocytes. Thus, the activity of minocycline seems to be unique in this group of antibiotics.

We showed that minocycline increased the intracellular level of melanin. The stimulating effect was observed after 3 h and 6 h from the irradiation. The level of melanin after minocycline treatment was higher than in melanocytes exposed to UVA radiation. However, UVB radiation appeared to be the most potent stimulator of melanin synthesis. Moreover, minocycline and both UV spectra showed a synergistic effect. Melanocytes simultaneously exposed to the drug and UV radiation had more melanin than unirradiated cells treated with minocycline. The highest melanin content was noticed in melanocytes exposed to the tested drug and UVB radiation (about 137% of control).

*MITF* is a key transcription regulator for melanogenesis, also termed as ‘master transcriptional regulator’ of the melanocyte lineage. It is responsible for the regulation of key genes for melanocyte development and differentiation [48]. It was found that the suppression of the *MITF* gene expression and degradation of the *MITF* protein or negatively regulation of its stability inhibited melanin synthesis [49,50].

The presented results revealed an increase in *MITF* expression caused by the treatment as well as the irradiation. The highest level of *MITF* was noticed in melanocytes exposed to UVA and UVB radiation. The observed increase in irradiated melanocytes was more than two-fold when compared to the control. The relative expression level of *MITF* in minocycline-treated cells was around 1.5. It was also found that the only UVB intensified the effect of minocycline. The Western blotting analysis indicated that the level of *MITF* was elevated in all tested samples and UVB appeared to be the most potent factor again. Moreover, only this spectrum additionally increased *MITF* content in cells treated with minocycline.

The direct influence of UV radiation on melanocytes and melanin synthesis is still not fully understood. The stimulation of melanogenesis by UV is mainly indirect under physiological conditions. The process is the result of UV-induced DNA damage in keratinocytes, which synthesize and excrete stimulating factors, including α-MSH [51]. However, previously conducted studies on melanocytes revealed some direct mechanisms. Wicks et al. proved the exposure of melanocytes to UVA radiation led to intensifying melanin synthesis by calcium mobilization and by the activation of retinal-dependent phototransduction pathway [52]. It was found that the pathway activation was mediated by transient receptor potential A1 (TRPA1) ion channels and led to melanocyte depolarization [53,54]. Moreover, Bellono et al. indicated that the UV-induced phototransduction mechanism in melanocytes involved the activation of a Gαq/11-dependent phosphoinositide cascade [55]. The presented results indicated that minocycline and UV radiation could directly influence melanocyte physiology and stimulated melanin synthesis. It was shown that both tested factors enhanced the transcriptional activity of the *MITF* and TYR genes. The expression of both genes was correlated; hence, it may suggest that induced hyperpigmentation might be via *MITF*-driven mechanism.

Tyrosinase gene is one of the targets for *MITF* transcription factor. The expression of tyrosine is observed exclusively in melanin-producing cells. Its gene encodes polypeptide involving an N-terminal signal peptide, a large intra-melanosomal domain, a single transmembrane α-helix and a small, flexible C-terminal cytoplasmic domain [56]. The glycosylation by N-linked glycans is a post-translational modification of tyrosinase. The process is necessary for proper polypeptide folding and is required for melanogenesis [57]. Tyrosinase is a copper-containing rate-limiting enzyme in the synthesis of melanin. The key role of the enzyme in melanin synthesis made tyrosinase a target for inhibitors of melanogenesis [58,59]. Previously it was founded that oxytetracycline and tetracycline did not affect the enzyme activity [46,47]. In turn, chlortetracycline and doxycycline at EC_50_ concentration had a tendency to inhibit tyrosinase in melanocytes [30,45]. The obtained results demonstrated that minocycline elevated the expression, protein level and activity of tyrosinase. These elevated levels were higher than observed for UVA and UVB radiation. Moreover, the synergistic action of minocycline and UV radiation was observed once again. The highest values for the protein level and tyrosinase activity were noticed for melanocytes exposed to the drug and UVB radiation simultaneously. Confocal microscopy imaging revealed that the intracellular distribution of tyrosinase in minocycline-treated melanocytes was similar to the disposal in UVB-irradiated cells. In addition, the enzyme appeared above cell nuclei after the treatment. The phenomenon was observed also in irradiated cells but not in control melanocytes. It suggests that the formation of supranuclear caps is not specific to UV radiation, but may also be caused by the side effects of a chemical factor.

Stimulation of melanogenesis by *MITF* transcription factor involves also transcriptional activation of non-enzymatic proteins. Pmel17 (also called gp100 or PMEL) is the most important of these proteins. It is involved in the formation of melanosomes at an early stage. Pmel17 creates amyloid fibrils that form a functional matrix in melanosomes. The matrix is responsible for the ellipsoidal shape and maturation of melanosomes as well as for the deposition of melanin pigment [60,61]. We showed that minocycline treatment decreased the level of Pmel17 protein in human melanocytes. The effect was also observed in cells simultaneously exposed to UV radiation. In turn, neither UVA nor UVB affected the protein level in non-treated cells significantly. The observation was surprising due to a different correlation in reference to results described above. Unexpectedly, minocycline appeared to be a disturbing factor for the formation of normal, mature melanosomes. It was stated that the depletion of Pmel17 led to the formation of enlarged and rounded melanosomes without fibrillar matrix [62]. Some studies indicated that the lack of Pmel17 was related to attenuation of melanogenesis and low viability of melanocytes [63,64]. Nevertheless, the role of Pmel17 has not been clearly and, finally, stated yet. Hellström et al. demonstrated that inactivation of the Pmel17 gene in a mouse line altered melanosome shape and melanin deposition; however, it had a little effect on visible pigmentation and coat color phenotype. Moreover, it was suggested that the lack of Pmel17 could reduce melanin transfer from melanocytes to keratinocytes [65]. Thus, in the face of these results, the disturbed distribution of melanin pigments can be one of the reasons for minocycline-induced darkly pigmented spots on the skin.

The obtained results revealed the molecular basis of type 3 minocycline-induced hyperpigmentation that is related to elevated melanin level, especially in sun-exposed areas of the skin. Taking into account the presented mechanisms, some potential preventive and therapeutic advice can be drawn. The indications reducing the risk or intensity of hyperpigmentation include adjustment of the daily dosage, a decrease of sun-exposure, wearing protective clothing and the use of sunscreen [7]. It is worth mentioning that minocycline-induced hyperpigmentation is reduced after discontinuation after the treatment [66]. Moreover, the use of lasers or tyrosinase-inhibiting substances may be considered to be a potentially effective therapy for this kind of skin discoloration [59,67,68].

## 4. Conclusions

To summarize, the presented results show that minocycline induces melanogenesis in human normal melanocytes. The effect is related to translational stimulation and the increased level of *MITF* and tyrosinase. It was observed that the melanin level and the activity of tyrosinase were significantly higher in treated cells than in control samples. The drug also caused changes in the intracellular distribution of tyrosinase. The supranuclear cumulation of the enzyme was observed, similar to cells exposed to UV radiation. Moreover, it was found that the action of minocycline was intensified by irradiation, especially with the UVB spectrum. To our surprise, the decreased level of Pmel17 was observed in all minocycline-treated melanocytes. The cell irradiation with UVA and UVB did not influence this action significantly. The finding suggests that the drug can disturb the formation, maturation, or distribution of melanosomes. However, further studies are necessary to solve this problem. Demonstrated results indicated the potential role of melanin biopolymers and the melanogenesis process minocycline-induced hyperpigmentation.

## 5. Materials and Methods

### 5.1. Chemicals and Reagents

Minocycline hydrochloride (C_23_H_27_N_3_O_7_ x HCl), Phosphatase Inhibitor Cocktail 3, SIGMAFAST Protease Inhibitor Cocktail Tablets, amphotericin B solution (250 µg/mL), penicillin, L-3,4-dihydroxyphenylalanine (L-DOPA), phosphate buffered saline (PBS), Tween-20, Tris(hydroxymethyl)aminomethane, RIPA Buffer and Phalloidin-Atto 565 were obtained from Sigma-Aldrich, Inc. (Taufkirchen, Germany). Neomycin sulphate was obtained from Amara (Kraków, Poland). Trypsin/EDTA was acquired from Cytogen (Zgierz, Poland). An M-254 growth medium and a human melanocyte growth supplement-2 (HMGS-2) were purchased from Cascade Biologics (Portland, OR, USA). Cell Proliferation Reagent WST-1 was obtained from Roche GmbH (Mannheim, Germany). Dulbecco’s phosphate-buffered saline (DPBS), DAPI (4′6-diamidino-2-phenylindole, dihydrochloride) 1 mg/mL in water, TRIzol Reagent, Pierce ECL Western Blotting Substrate and Tyrosinase (T311) monoclonal antibody as well as secondary antibody-Alexa Fluor 488 were purchased from Thermo Fisher Scientific, Inc. (Waltham, MA, USA). Via-1-Cassettes™ (acridine orange and DAPI fluorophores) were obtained from ChemoMetec (Lillerød, Denmark). Pmel17 (E7) monoclonal antibody was acquired from Santa Cruz Biotechnology, Inc. (Dallas, TX, USA). GAPDH (14C10) and *MITF* (D5G7V) monoclonal antibodies were obtained from Cell Signaling (Danvers, MA, USA). Fluorescence mounting medium was purchased from Agilent Dako (Carpinteria, CA, USA). The remaining reagents and chemicals were produced by POCH SA (Gliwice, Poland).

### 5.2. Cell Culture

Human epidermal melanocytes, neonatal, darkly pigmented HEMn-DP were obtained from Cascade Biologics, USA. The melanocytes used in the study are primary cells isolated from one neonatal donor foreskin.

All in vitro studies were performed on melanocytes from passages 6 to 10. The growth medium was supplemented with a human melanocyte growth supplement-2 (HMGS-2) as well as antibiotics: penicillin (100 U/mL), neomycin (10 μg/mL) and amphotericin B (0.25 μg/mL). Melanocytes were cultured in a 5% CO_2_ incubator CB 160 (BINDER, Tuttlingen, Germany) at 37 °C with 5% relative humidity.

### 5.3. Melanocyte Treatment

Normal human melanocytes were seeded in Petri dishes (1×10^6^ cells/dish) or in a 96-well microplate (5×10^3^ cells/well) and incubated with the growth medium for 48 h. Then, the medium was replaced with minocycline solutions in the growth medium. At the same time, the medium was changed in non-treated samples. After one day treatment, some samples were irradiated with UVA (λ_max_ = 365 nm, 2 J/cm^2^, 720 µW/cm^2^) and UVB (λ_max_ = 312 nm, 10 mJ/cm^2^, 600 µW/cm^2^) radiation using a lamp BVL-8.LM (Vilber Lourmat, Marne-la-Vallée, France). The irradiation was performed after the medium and minocycline solutions were replaced by PBS. After irradiation, all tested cell culture were incubated with the growth medium up to analysis.

### 5.4. Screening Test of Cells Proliferation

Melanocyte proliferation was tested by the WST-1 assay. WST-1 is a slightly red tetrazolium salt that can be reduced to dark red formazan dye by mitochondrial dehydrogenases in viable cells. The experiment was conducted according to the Melanocyte treatment procedure using 96-well microplate. WST-1 reagent was added to the tested culture in an amount of 10 µl/well 21 h after irradiation. The measurement was made after 3 h using microplate reader Infinite 200 PRO (TECAN, Männedorf, Switzerland). Absorbance readings were taken at 440 nm and 650 nm as a reference wavelength. Control samples were normalized to 100% and all tested samples were calculated as the percentage of the control.

### 5.5. The Assessment of Cell Number and Cell Viability

Cell counting and the estimation of cell viability were made using a fluorescent imaging cytometer NucleoCounter^®^ NC-3000™ (ChemoMetec). The method is based on staining of non-fixed cells with acridine orange (detection of total cells population) and DAPI (detection of dead cells). Melanocytes were harvested 6 h after irradiation. Then, they were centrifuged, resuspended in the growth medium and loaded into Via1-Cassettes™ (ChemoMetec) containing the stains. Then, the cells were immediately analyzed using the Cell Viability and Cell Count Assays protocol by an NC-3000 image cytometer. The instant and rapid analysis means that DAPI penetrates a damaged and permeable cell membrane; hence, it only stains non-viable cells in this condition.

### 5.6. Real-Time Quantitative PCR Analysis

The total RNA was extracted from melanocytes using TRIzol Reagent. Next, melanin polymers were removed from RNA extracts by the use OneStep™ PCR Inhibitor Removal Kit (Zymo Research, Irvine, CA, USA). The total amount of RNA was measured using a microvolume spectrophotometer DS-11 (DeNovix^®^, Wilmington, DE, USA). Quantitative RT-PCR analysis was performed using SensiFAST™ SYBR No-ROX kit (Meridian Bioscience, Cincinnati, OH, USA) and specific primers for *MITF*, *TYR* and *GAPDH* genes (Sigma-Aldrich Inc., Germany). The primer sequences were presented in Table 1. The measurement was made using LightCycler^®^ 480II (Roche, Penzberg, Germany) according to the following reaction parameters: 45 °C for 10 min, 95 °C for 2 min, 45 cycles of 95 °C for 5 s, 60 °C for 10 s, 72 °C for 1 min and the final extension for 10 min at 72 °C. The mRNA levels were calculated using the 2^−ΔΔCt^ method. The obtained results were normalized using *GAPDH* expression and converted into expression level relative to the control.

### 5.7. Preparation of Cell Lysates

Tested melanocytes were suspended in a lysis buffer 3 h and 6 h after the irradiation. The lysis buffer contained a phosphatase inhibitor (10 µL/mL) and a protease inhibitor (1.4 mg/mL) dissolved in PBS. The lysates were prepared by freezing cells in liquid nitrogen (−196 °C). Melanocyte lysates were stored at −86 °C until the analysis of total protein concentration, melanin content and activity of tyrosinase.

### 5.8. The Analysis of Protein Concentration

The concentration of total protein in cell lysates was determined using Pierce™ BCA Protein Assay Kit (Thermo Fisher Scientific Inc., USA), according to the producer instruction. The assay is based on the ability of protein to reduction of Cu^2+^ to Cu^1+^ in an alkaline medium as well as the selective and sensitive colorimetric detection of Cu^1+^ by bicinchoninic acid (BCA). The measurement of absorbance was performed at 562 nm using a microvolume spectrophotometer DS-11 (DeNovix, Wilmington, DE, USA).

### 5.9. The Analysis of Melanin Content

The evaluation of melanin content was carried out after 1 h heating of cell lysates with 1 M NaOH at 80 °C. Prepared samples were centrifuged for 20 min at 16,000× *g* before analysis. The spectrophotometric measurement of supernatants was made at 405 nm using microplate reader Infinite 200 PRO (TECAN, Männedorf, Switzerland). The obtained results were normalized using total protein concentration and expressed as the percentage of control.

### 5.10. The Analysis of Tyrosinase Activity

Spectrophotometric determination of tyrosinase activity was based on the enzymatic oxidation of L-DOPA to a colorful DOPAchrome. A solution of tyrosinase substrate (L-DOPA) was prepared in the phosphate buffer (pH = 6.8) at a concentration of 2 mg/mL. Melanocyte lysate were clarified by centrifugation at 10,000× *g* for 5 min. The obtained supernatants in an amount of 100 μL and 40 μL of L-DOPA solution were placed in a 96-well plate. Then, the absorbance of dopachrome was measured at 475 nm every 10 min for at least 1.5 h at 37 °C using a microplate reader Infinite 200 PRO (TECAN, Männedorf, Switzerland). The obtained results were normalized using total protein concentration and expressed as the percentage of control.

### 5.11. Western Blotting Analysis

The examined cells were lysed 6 h after the irradiation procedure. Cell lysis were made using RIPA buffer containing phosphatase and protease inhibitors. Prepared lysates were centrifuged at 12,000 rpm for 10 min at 4 °C to purification of lysates from melanin stored at −86 °C until assessment of total protein concentration and Western blotting analysis. Protein extracts (20 µg/lane) were separated on an 10% SDS-polyacrylamide gel electrophoresis and transferred to PVDF membranes (Sigma-Aldrich, Inc., USA). Afterward, the membranes were incubated for 1 h in blocking buffer (a solution of 5% non-fat milk and TBST: Tris-buffered saline with Tween 20) and washed with TBST. The analyzed proteins were detected using primary monoclonal antibodies: rabbit anti-GAPDH (1:1,000), mouse anti-TYR (1:100), rabbit anti-*MITF* (1:500), mouse anti-Pmel17 (1:500) diluted in blocking buffer. The incubation was carried out overnight at 4 °C. Next, the membranes were washed with TBST and incubated for 1.5 h at room temperature with appropriate horseradish peroxidase-conjugated secondary antibody (1:10,000) diluted in blocking buffer. Finally, the protein signals were detected using ECL reagent. The analysis was made using G:Box Chemi-XT4 Imaging System and GeneTools Software (Syngene, Cambridge, UK). The obtained results were normalized using the level of GAPDH and expressed as the percentage of control.

### 5.12. Microscopic Assessment of Melanocytes

Imaging of darkly pigmented human melanocytes were performed using light inverted microscope NIKON TS100F (Japan) as well as the laser confocal microscope Nikon Eclipse Ti-E A1R-Si and Nikon NIS Elements AR software. The cells were cultured in sterile cover slips placed in Petri dishes for 48 h. Next, the cells were treated with minocycline and exposed to UVA and UVB radiation, according to paragraph 5.3. The exanimated cells were fixed with paraformaldehyde (4%) and permeabilized with 0.1% Triton X-100 6 h after the irradiation procedure. All samples were blocked with glycine and BSA solutions and then were incubated with primary anti-tyrosinase antibody (1:100) overnight at 4 °C. Afterward, melanocytes were stained with DAPI (1:500), Phalloidin–Atto 565 (0.6 µM) and conjugated with the secondary antibody-Alexa Fluor 488 (1:200). The staining allowed to image nucleus, actin filaments and tyrosinase, respectively. Finally, the cover slips were mounted onto microscopic glass slides.

### 5.13. Statistical Analysis

Statistical analysis of the results was performed using GraphPad Prism 6.01 Software. In all the experiments, mean values of at least three separate experiments performed in triplicate (*n* = 9) ± standard deviation of the mean (SD) were calculated. The results were analyzed statistically by one-way ANOVA and two-way ANOVA, as well as Dunnett’s and Tukey’s multiple comparison tests. The Kolmogorov–Smirnov test checked the compliance of the distribution results and the Brown–Forsythe test checked the variances of the compared groups meet the homogeneity assumption. In all cases, the statistical significance was found for the *p*-value to be lower than 0.05.

## Figures and Tables

**Table 1:** Nucleotide sequences of primers used in RT-qPCR analysis.

Gene	Forward Primer (5′→3′)	Reverse Primer (5′→3′)
*MITF*	CAGTACCTTTCTACCACTTTAG	CCTCTTTTTCACAGTTGGAG
*TYR*	CAACAGCCATCAGTCTTTATG	CCTTCCAGTGTATTTCTAAAGC
*GAPDH*	CTTTTGCGTCGCCAG	TTGATGGCAACAATATCCAC

## Data Availability

The data presented in this study are available on request from the corresponding author.
